# Exosomal circRNA_100284 from arsenite-transformed cells, via microRNA-217 regulation of EZH2, is involved in the malignant transformation of human hepatic cells by accelerating the cell cycle and promoting cell proliferation

**DOI:** 10.1038/s41419-018-0485-1

**Published:** 2018-04-19

**Authors:** Xiangyu Dai, Chao Chen, Qianlei Yang, Junchao Xue, Xiong Chen, Baofei Sun, Fei Luo, Xinlu Liu, Tian Xiao, Hui Xu, Qian Sun, Aihua Zhang, Qizhan Liu

**Affiliations:** 1 Institute of Toxicology, School of Public Health, Nanjing Medical University, Nanjing, 211166 Jiangsu People’s Republic of China; 2 The Key Laboratory of Modern Toxicology, Ministry of Education, School of Public Health, Nanjing Medical University, Nanjing, 211166 Jiangsu People’s Republic of China; 3 The Key Laboratory of Environmental Pollution Monitoring and Disease Control, Ministry of Education, School of Public Health, Guizhou Medical University, Guiyang, 550025 Guizhou People’s Republic of China

## Abstract

Intercellular communication between malignant cells and neighboring nonmalignant cells is involved in carcinogenesis. In the progression of carcinogenesis, exosomes are messengers for intercellular communication. Circular RNAs (circRNAs) are noncoding RNAs with functions that include regulation of the cell cycle and proliferation. However, the functions of exosomal circRNAs are not clear. The present research aimed to determine whether circRNAs secreted from arsenite-transformed human hepatic epithelial (L-02) cells are transferred into normal L-02 cells and become functionally active in the normal cells. The results showed that circRNA_100284 is involved in the malignant transformation of L-02 cells induced by arsenite. The medium from transformed L-02 cells induced upregulation of circRNA_100284, accelerated the cell cycle, and promoted proliferation of normal L-02 cells. Transformed cells transferred circRNA_100284 into normal L-02 cells via exosomes and led to the malignant transformation of the non-transformed cells. Knockdown of circRNA_100284, which reduced circRNA_100284 levels in exosomes derived from transformed L-02 cells, blocked the accelerated cell cycle and reduced proliferation and malignancy. In addition, in normal L-02 cells, exosomal circRNA_100284 derived from arsenite-transformed L-02 cells induced acceleration of the cell cycle and promoted proliferation via acting as a sponge of microRNA-217. Further, exosomal circRNA_100284 was upregulated in the sera of people exposed to arsenite. Thus, exosomes derived from transformed L-02 cells transferred circRNA_100284 to surrounding cells, which induced an accelerated cell cycle and promoted proliferation of normal liver cells and led to the malignant transformation of the non-transformed cells. The findings support the concept that exosomal circRNAs are involved in cell–cell communication during carcinogenesis induced by arsenite.

## Introduction

Arsenic is a naturally existing, toxic metalloid that is often a contaminant in drinking water, and there can be harmful effects from arsenic even when levels are below the drinking water standard. Long-term exposure to arsenic is associated with lung, bladder, and skin cancer, and with noncancerous disorders such as cardiovascular disease, diabetes, and skin lesions^1,2^. Carcinogenesis induced by arsenic is related to genetic variants^3,4^, oxidative DNA damage^5^, and DNA methylation^6^. Inorganic arsenic affects various signaling pathways^7^ and stimulates cell proliferation by increasing the population of cells in the S phase of the cell cycle^8^. However, the molecular mechanisms remain unclear.

Noncoding RNAs (ncRNAs) are not translated into protein. Abundant and functionally active types of noncoding RNAs include transfer RNAs (tRNAs), ribosomal RNAs (rRNAs), small RNAs such as microRNAs (miRNAs), and long ncRNAs (lncRNAs). Chronic exposure to arsenite causes abnormal expression of various ncRNAs, including miR-21;^9^ miR-143;^10^ and the lncRNA MALAT1^11^.

Circular RNA (circRNA), unlike the better-known linear RNA, has a covalently closed, continuous loop, with the 3’ and 5’ ends joined together. For humans, thousands of circRNAs have been identified by use of p(A)-without RNase R RNA-seq data^12^. Several circRNAs serve as biomarkers in the development of hepatocellular carcinomas and regulate gene expression by acting as “miRNA sponges”^13,14^. By use of a circRNA microarray, we found, in a previous study, that, in arsenite-transformed HaCaT cells, circRNA_100284 showed the greatest upregulation and was involved in the arsenite-accelerated cell cycle of human keratinocytes in the process of carcinogenesis^15^.

In the tumor microenvironment, crosstalk of malignant cells with nonmalignant cells is essential for tumor progression^16^. Exosomes, with diameters of 30–100 nm, are cell-derived vesicles that are present in many, and perhaps all, eukaryotic fluids, including blood, and urine, and in the culture medium of cells^17,18^. Exosomes carry messenger RNAs (mRNAs), miRNAs^19^, and double-stranded DNA^20^. Exosomes are involved in cell-to-cell signaling and may influence processes in normal cells because they can merge with and release their contents into cells that are distant from their cell of origin. For example, as shown in our previous studies, RNAs shuttled from one cell to another, known as “exosomal shuttle RNAs,” can affect protein production in normal cells^21,22^. In exosomes, circRNAs are enriched and stable^23^, and, in KRAS mutant colon cancer cells, circRNAs can be transferred into exosomes^24^. However, the functions of exosomal circRNAs remain undefined.

In the present study, we investigated the influence of arsenic-transformed L-02 cells on the cell cycle and cell proliferation of normal liver cells. Chronic exposure to arsenite elevated circRNA_100284 levels, which were involved in the malignant transformation of normal L-02 cells. circRNA_100284 was contained in exosomes released by transformed cells and could be transferred into normal cells, and exosomal circRNA_100284 regulated the cell cycle and stimulated cell proliferation of normal liver cells by interacting with miRNA-217 (miR-217). These findings contribute to an understanding of the processes involved in carcinogenesis caused by arsenite.

## Materials and methods

### Cell culture and reagents

L-02 cells, a line of normal human liver cells, were obtained from the Shanghai Institute of Cell Biology, Chinese Academy of Sciences (Shanghai, China) and were maintained in 5% CO_2_ at 37 °C in RPMI-1640 medium supplemented with 10% fetal bovine serum (FBS, Life Technologies/Gibco, Grand Island, NY), 100 U/mL penicillin, and 100 μg/mL streptomycin (Life Technologies/Gibco, Gaithersburg, MD). We previously established the model of arsenite-transformed L-02 cells^25^. For chronic exposure, 1 × 10^6^ L-02 cells were seeded into 10-cm (diameter) dishes for 24 h and maintained in 0 or 2 μM sodium arsenite (NaAsO_2_, Sigma, St. Louis, MO; purity, 99.0%) for 48–72 h per passage. This process was continued for ~15 weeks (30 passages).

### Nude mouse tumorigenicity assay

Female athymic nude mice (5 weeks old) were purchased from the Animal Research Center of Nanjing University (China) and housed in a specific pathogen-free room in the Animal Facility at the Nanjing Medical University. Animals were treated humanely and with regard for alleviation of suffering according to a protocol approved by the Nanjing Medical University Institutional Animal Care and Use Committee. Mouse xenograft assays were performed as described previously^26^. Briefly, 1 × 10^7^ cells were injected subcutaneously into the right armpits of nude mice. There were six mice per group. Tumor incidence and size were monitored once per week. After 4 weeks, tumor tissues were removed. Tumor volume was calculated by the modified ellipsoid formula:^27^ tumor volume (mm^3^) = 1/2 (*xy*^2^), where *x* is the greatest longitudinal diameter and *y* is greatest transverse diameter.

### Quantitative real-time PCR

Total cellular RNA was isolated by Trizol (Invitrogen) according to the manufacturer’s recommendations. To detect circRNAs and miRNAs, 2 μg of total RNA and HiScript II Q Select RT Supermix (Vazyme Biotech) were used in reverse transcription according to the manufacturer’s protocol. For detection of circRNA_100284, GAPDH RNA was used as a control. Forward (F) and reverse (R) primers, synthesized by Invitrogen, were as follows: circRNA_100284-F: 5’-A*CTCACAATGATCCAAAAGGAGT*-3’; circRNA_100284-R: 5’-*GAGATACAGTGCATTCCAAGACA*-3’; GAPDH-F, 5’-*GCATCCTGGGCTACACTG*-3’; GAPDH-R, 5’-*TGGTCGTTGAGGGCAAT*-3’. Quantitative real-time PCR was performed with Power SYBR Green Master Mix (Applied Biosystems, Foster City, CA, USA) and a Roche LightCycler96 machine. Fold changes in expression of each gene were calculated by a comparative threshold cycle (Ct) method using the formula 2^−(ΔΔCt)^^28^.

### Western blots

Cell lysates were prepared with a detergent buffer as described previously^25^. Protein concentrations were measured with the BCA Protein Assay according to the manufacturer’s manual (Beyotime Institute of Biotechnology, Shanghai, China). Equal amounts (80 μg) of protein were separated by 10% sodium dodecyl sulfate-polyacrylamide gel electrophoresis and were transferred to polyvinylidene fluoride membranes (PVDF, Millipore, Billerica, MA). Membranes were incubated overnight at 4 °C with a 1:1000 dilution of anti-tubulin (Abcam) and antibodies for EZH2 or cyclin-D1 (Cell Signaling Technology, Beverly, MA). After additional incubation with a 1:1000 dilution of an anti-immunoglobin horseradish peroxidase-linked antibody for 1 h, the immune complexes were detected by enhanced chemiluminescence (Cell Signaling Technology). For densitometric analyses, protein bands on the blots were measured by the use of Image J software.

### Exosome isolation

The culture medium was collected and centrifuged at 3000×*g* for 15 min, and the supernatant was filtered through a 0.22-mm PVDF filter (Millipore). An appropriate volume of Exoquick exosome precipitation solution (System Biosciences) was added to the filtered culture medium, or a quarter volume of Exoquick exosome precipitation solution was added to the serum. After mixing and refrigeration for 24 h, the mixture was centrifuged at 1500×*g* for 30 min, and the supernatant was removed. Exosome pellets from 1 × 10^6^ cells were suspended in 150 µL of serum-free medium. The size distribution and concentration of exosomes were analyzed by nanoparticle-tracking analysis using a ZetaView particle tracker from ParticleMetrix (Germany).

### Transmission electron microscopy (TEM)

The exosome-enriched pellets were suspended in 50 mL of PBS, fixed with 4% paraformaldehyde and 4% glutaraldehyde in 0.1 M phosphate buffer (pH 7.4) at incubation temperature and kept at 4 °C until analysis by TEM. A drop of each exosome sample was placed on a carbon-coated copper grid and immersed in 2% phosphotungstic acid solution (pH 7.0) for 30 s. The preparations were examined with a transmission electron microscope (JEM-1200EX; JEOL Ltd., Tokyo, Japan) at an acceleration voltage of 80 kV.

### Exosome labeling

Exosomes from 1.5 × 10^6^ cells were suspended in 180 μL of PBS with 20 μL of 1:50 diluted PKH67 (Sigma, in Diluent C). After 3 min of incubation at room temperature (RT), 3.8 mL of exosome-free medium was added to terminate the labeling reaction, and exosomes were harvested and washed twice with PBS by centrifugation (100,000 g for 1 h). Exosomes were suspended in 9.6 mL of basal medium, and 250 μL was added to a sub-confluent layer of L-02 or T-L-02 cells, which were incubated for 3 h at 37 °C. Cells were washed twice with PBS and fixed with 4% paraformaldehyde in PBS for 30 min at RT. To stain the nuclei, 4′,6-diamidino-2-phenylindole (DAPI, Sigma) was added for 10 min, and stained cells were observed under a fluorescence microscope (Zeiss, LSM700B, Germany).

### Cell transfection

An miR-217 mimic, an miR-217 inhibitor, an miRNA negative control mimic (con mimic), an miRNA negative control inhibitor (con inhibitor), a circRNA_10084 siRNA and a control siRNA were synthesized by RiBoBio (Guangzhou, China). A pLCDH- circRNA_10084, and a pLCDH control were synthesized by GENESEED (Guangzhou, China). Cells were transiently transfected by use of Lipofectamine 2000 reagent (Invitrogen) according to the manufacturer’s protocol. At 24 h after transfection, cells were harvested and used for experiments.

### Flow cytometry

L-02 cells were treated with culture medium or exosomes isolated from arsenite-transformed L-02 cells that were transfected with circRNA_100284 siRNA or control siRNA, and an miR-217 inhibitor or control inhibitor for 24 h, and the cells were harvested after 24 h. After washing twice with cold PBS, cells were fixed with 75% ethanol at 4 °C overnight and again washed twice with cold PBS. After treatment with RNase A at 37 °C for 30 min and staining with propidium iodide, 10^5^ nuclei were examined with a FACS Calibur flow cytometer (Becton Dickinson, USA). The results were presented as the percentages of cells in each phase.

### Determination of cell proliferation

Cell proliferation was evaluated by WST-8 hydrolysis using Cell Counting Kit-8 (Dojindo Molecular Technologies, Inc.) as described previously^29^. Briefly, cells were seeded into 96-well tissue culture plates at 4000 cells per well. Plates were incubated for 0, 12, 24, 48, 72, or 96 h at 37 °C with 5% CO_2_ in a humidified incubator. After cells were treated and after incubation, 20 μL of WST-8 was added to each well and the incubation continued for an additional 3 h. Plates were read on a Biorad ELISA plate reader (BioTEK Instruments, Winooski, VT, USA) using a 450-nm filter. Results of at least three independent experiments were analyzed in duplicate. The relative cell proliferation ratios were plotted along with non-treated controls to determine the 100% level of activity.

### Anchorage-independent growth

Soft agar dishes were prepared with under layers of 0.70% agarose in 1640 medium supplemented with 10% FBS. To test their capacity for colony growth in soft agar, treated L-02 cells were plated in triplicate at a density of 1 × 10^4^ in 2 mL of 0.35% agarose over the agar base. Cultures were fed every 3 days. After ~14 days, the colonies were observed under a microscope, and those with diameters >80 μm were counted. These represent colonies with >30 cells.

### Transwell assays

Migration of arsenite-transformed L-02 cells was evaluated by use of Transwell chambers with 8-μm pore filters (Corning Inc., Corning, NY, USA). At 24 h after transfection, cells (5 × 10^4^/100 μL) were plated on the upper chambers in serum-free medium; 1640 medium containing 10% FBS was added to the lower chambers as a chemoattractant. After incubation for 24 h at 37 °C, non-migrating cells were removed with cotton swabs. Cells that migrated to the bottom of the membrane were fixed with 4% paraformaldehyde, stained with crystal violet solution for 30 min, and washed twice with PBS. Stained cells were visualized under a microscope (high-power field), and the numbers of cells counted in five random fields were averaged. To assess the capacity for invasion of arsenite-transformed L-02 cells, transfected cells (5 × 10^4^/100 μL) were added to upper chambers that had been coated with 35 μL of Matrigel (BD Biosciences, Franklin Lakes, NJ, USA); 1640 medium containing 10% FBS was added to the lower chambers. Cells were incubated for 24 h at 37 °C, and non-invading cells were removed with cotton swabs. Invading cells were fixed, stained, and counted.

### Statistical analyses

Derived values are presented as means ± SD. Comparisons of means among multiple groups were performed by one-way analysis of variance (ANOVA), and a multiple-range least significant difference was used for intergroup comparisons. *P* values < 0.05 were considered statistically significant. All statistical analyses were performed with SPSS 16.0.

## Results

### Effects of circRNA_100284 on the neoplastic capacity of arsenite-transformed L-02 cells

**Fig. 1:**
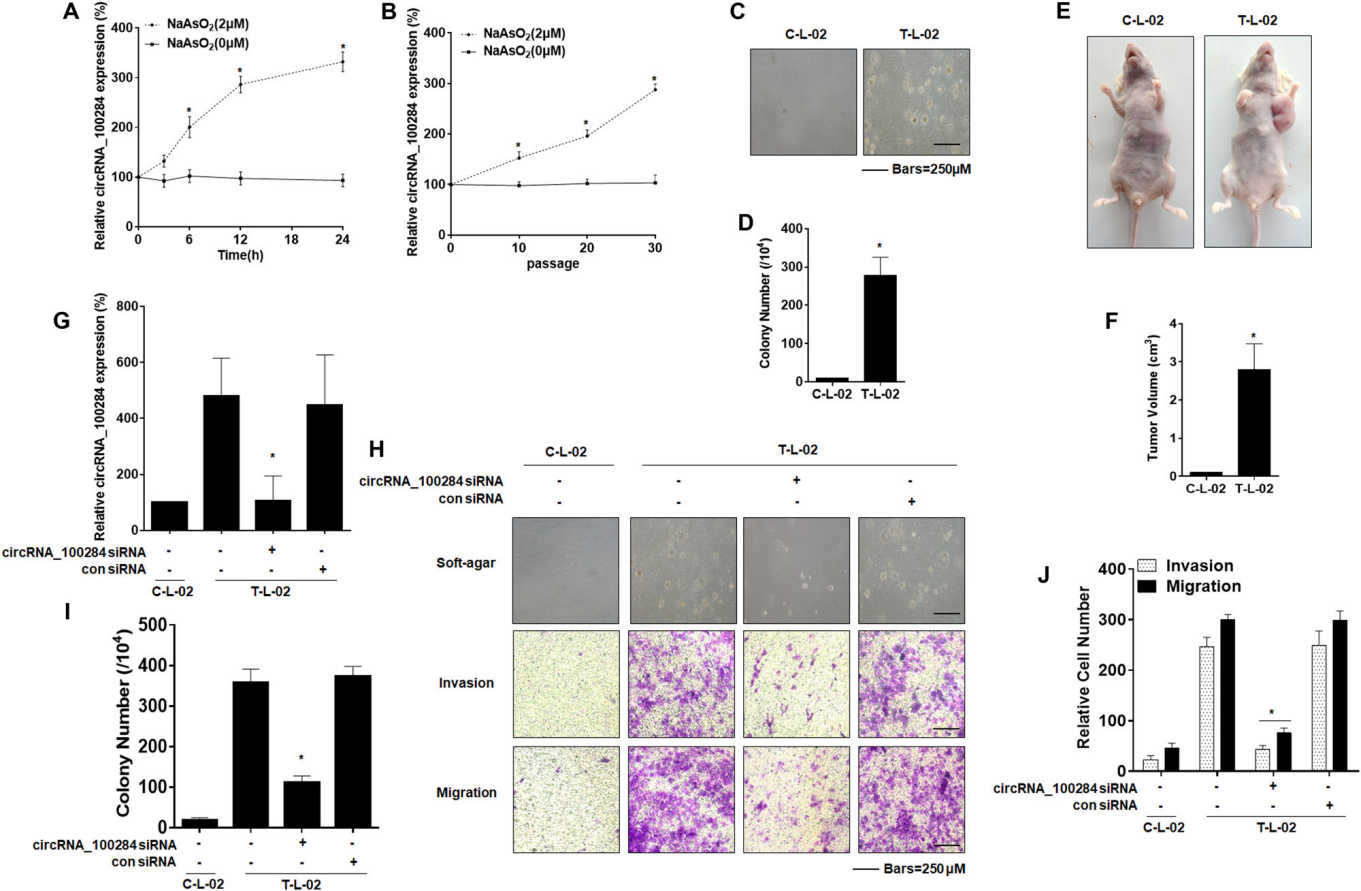
Effects of circRNA_100284 on the neoplastic capacity of arsenite-transformed L-02 cells. *C-L-02*, passage-control L-02 cells; *T-L-02*, arsenite-transformed L-02 cells. L-02 cells were treated with 0 or 2 μM of arsenite for 0, 3, 6, 12, or 24 h. **a** The levels of circRNA_100284 were determined by qRT-PCR assays (means ± SD, *n* = 3). L-02 cells were exposed to 0 or 2 μM of arsenite for 0, 10, 20, or 30 passages. **b** The levels of circRNA_100284 were determined by qRT-PCR assays (means ± SD, *n* = 3). *P < 0.05, different from control cells. **c** Colonies and (**d**) their numbers (means ± SD, *n* *=* 3) of C-L-02 cells and T-L-02 cells in soft agar (bars = 250 μm). **e** Tumors were examined, and (**f**) their volumes were measured (means ± SD, *n* *=* 6) at 4 weeks after C-L-02 cells and T-L-02 cells were inoculated into nude mice. *P < 0.05, different from C-L-02 cells. T-L-02 cells were transfected with 10 nM control siRNA or 10 nM circRNA_100284 siRNA for 24 h. **g** The levels of circRNA_100284 were determined by qRT-PCR assays (means ± SD, *n* = 3). **h** Colony formation in soft agar (upper, bars = 250 μm) and Transwell assays (lower, bars = 250 μm). **i** and (**j**) relative colony numbers and relative levels of cell invasion and migration were determined (means ± SD, *n* = 3). *P < 0.05, different from T-L-02 cells

To investigate whether circRNA_100284 was associated with arsenite-induced malignant transformation of liver epithelial cells, L-02 cells were treated with 0 or 2 μM of arsenite for 0, 3, 6, 12, or 24 h. Real-time PCR revealed that levels of circRNA_100284 were increased by arsenite in a time-dependent manner (Fig. 1a). Furthermore, L-02 cells were exposed to 0 or 2 μM of arsenite for 0, 10, 20, or 30 passages. With longer times of exposure to arsenite, the levels of circRNA_100284 showed a rising trend, which was not obvious for control cells (Fig. 1b). To establish whether L-02 cells were transformed after long exposures to arsenite, the anchorage-independent growth capacity of arsenite-transformed L-02 cells was assessed. L-02 cells formed colonies in agar after exposure to 2 μM arsenite for 30 passages; control cells did not show this capacity (Figs. 1c,d). Moreover, the tumor incidence in the group of mice injected with arsenite-transformed L-02 cells was 100% (6/6 per group); for the control group, the incidence was 0% (0/6). The tumor volumes for the group implanted with arsenite-transformed cells were determined (Figs. 1e,f). These results indicate that exposure to arsenite induces the malignant transformation of normal liver cells, accompanied with the overexpression of circRNA_100284.

Since circRNA_100284 was upregulated in arsenite-transformed L-02 cells, the influence of circRNA_100284 on arsenite-induced malignancy was examined. We first targeted circRNA_100284 using siRNAs specifically against the back-splice junction of circRNA_100284. Three siRNAs were designed for screening. The proper siRNA should have the capacity to knock down the circRNA_100284 without affecting the levels of its linear isoform, GCLM mRNA. siRNA#3 matched these criteria (Figs. S1A and S1B) and was used in further research. Determined by qRT-PCR, the level of circRNA_100284 decreased after transfecting cells with siRNA compared with that in arsenite-transformed L-02 cells (Fig. 1g). There was only a slight recovery in circRNA_100284 expression (Fig. S1C). After 14 days of incubation under anchorage-independent conditions, arsenite-transformed L-02 cells transfected with circRNA_100284 siRNA formed fewer colonies in agar than those transfected with control siRNA (Fig. 1h, upper, and Fig. 1i). In addition, silencing of circRNA_100284 in arsenite-transformed cells inhibited cell invasion and migration (Fig. 1h, lower, and Fig. 1j). Moreover, to investigate the malignancy of L-02 cells that had been exposed to 2 μM arsenite for 20 passages (As-L-02-p20 cells), a test for soft agar colony formation and a xenograft study were performed. Since the results showed no differences in numbers of colonies (Fig. S2A and Fig. S2B) or sizes of tumors (Fig. S2C and Fig. S2D) compared to the passage-control normal L-02 cells, the As-L-02-p20 cells could be considered as non-transformed cells. Moreover, we overexpressed circRNA_100284 in the As-L-02-p20 cells. qRT-PCR showed that the expression of circRNA_100284 was upregulated after transfection with pLCDH- circRNA_100284 (Fig. S2E). As-L-02-p20 cells transfected with pLCDH- circRNA_100284 formed more colonies in agar than those transfected with the pLCDH control (Fig. S2F, upper, and Fig. S2G). In addition, overexpression of circRNA_100284 led to enhanced invasion and migration (Fig. S2F, lower, and Fig. S2H). Thus, circRNA_100284 is involved in the neoplastic and metastatic capacity of arsenite-transformed L-02 cells.

### Medium from arsenite-transformed L-02 cells induces circRNA_100284 upregulation, acceleration of the cell cycle, and proliferation of normal L-02 cells

**Fig. 2:**
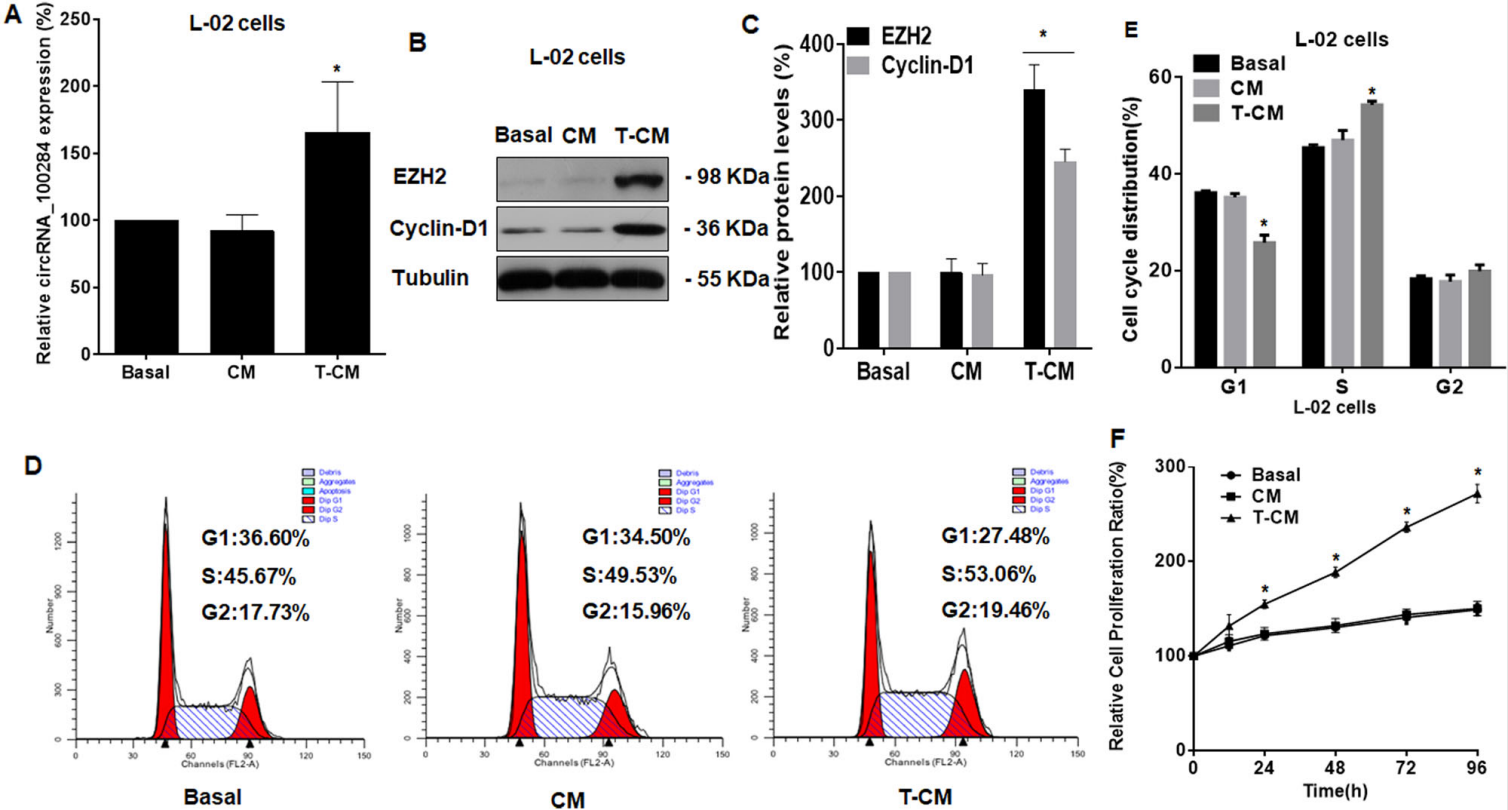
Medium from arsenite-transformed L-02 cells induces increases of circRNA_100284 levels, a disorder of the cell cycle, and proliferation in normal L-02 cells. *Basal* basal medium, *CM* medium from passage-control L-02 cells, *T-CM* medium from arsenite-transformed L-02 cells. Densities of bands were quantified by Image J software. Tubulin levels, measured in parallel, served as controls. L-02 cells were treated with various conditioned media. L-02 cells were cultured with Basal, CM, or T-CM for 24 h. **a** circRNA_100284 levels in L-02 cells were determined by qRT-PCR assays (means ± SD, *n* = 3). **b** Western blots were performed, and (**c**) relative protein levels (means ± SD, *n* = 3) of EZH2 and cyclin-D1 were determined in L-02 cells. **d** Flow cytometry was performed to analyze the cell cycle, and (**e**) representative histograms were prepared. **f** Proliferation efficiency was measured by use of a Cell Counting Kit-8 assay, and the relative ratios of cell proliferation were determined by comparison with cells in basal medium (means ± SD, *n* = 3). *P < 0.05, different from cells in basal medium

Interactions between cancer cells and neighboring cells in the extracellular environment are involved in the progression of the epithelial–mesenchymal transition and promote cancer development and metastasis^30,31^, we presumed that circRNA_100284 levels in normal cells were affected by transformed cells through intercellular communication. To prove this hypothesis, normal L-02 cells were exposed to basal medium, medium from passage-control L-02 cells, or medium from arsenite-transformed L-02 cells for 24 h. The levels of circRNA_100284 in normal L-02 cells were elevated when they were exposed to medium from arsenite-transformed L-02 cells relative to the basal medium or medium from passage-control L-02 cells (Fig. 2a).

Deregulated cell proliferation resulting from altered cell cycle progression is a main event in malignant and neoplastic transformation^32^. Elevated expression of enhancer of zeste homolog 2 (EZH2) in cell lines regulates the cell cycle via cyclin-D1 and increases proliferation and oncogenic capacity^33,34^. The levels of EZH2 and cyclin-D1 in normal L-02 cells treated with various media were measured. Normal L-02 cells treated with medium from arsenite-transformed L-02 cells showed elevated levels of EZH2 and cyclin-D1 (Figs. 2b,c). In addition, flow cytometry showed that the cell cycle was accelerated in cells treated with the medium from arsenite-transformed L-02 cells, as determined by the accelerated G1/S transition (Figs. 2d,e). Furthermore, the medium from arsenite-transformed L-02 cells promoted the growth of normal cells at multiple time points (24, 48, 72, and 96 h) relative to the growth of cells treated with basal medium (Fig. 2f). These results showed that, for normal liver cells, medium from arsenite-transformed L-02 cells induced increases of circRNA_100284 levels, an accelerated cell cycle, and abnormal cell proliferation.

### Exosomes derived from arsenite-transformed L-02 cells are transferred into normal L-02 cells and induced increases of circRNA_100284 in normal L-02 cells

**Fig. 3:**
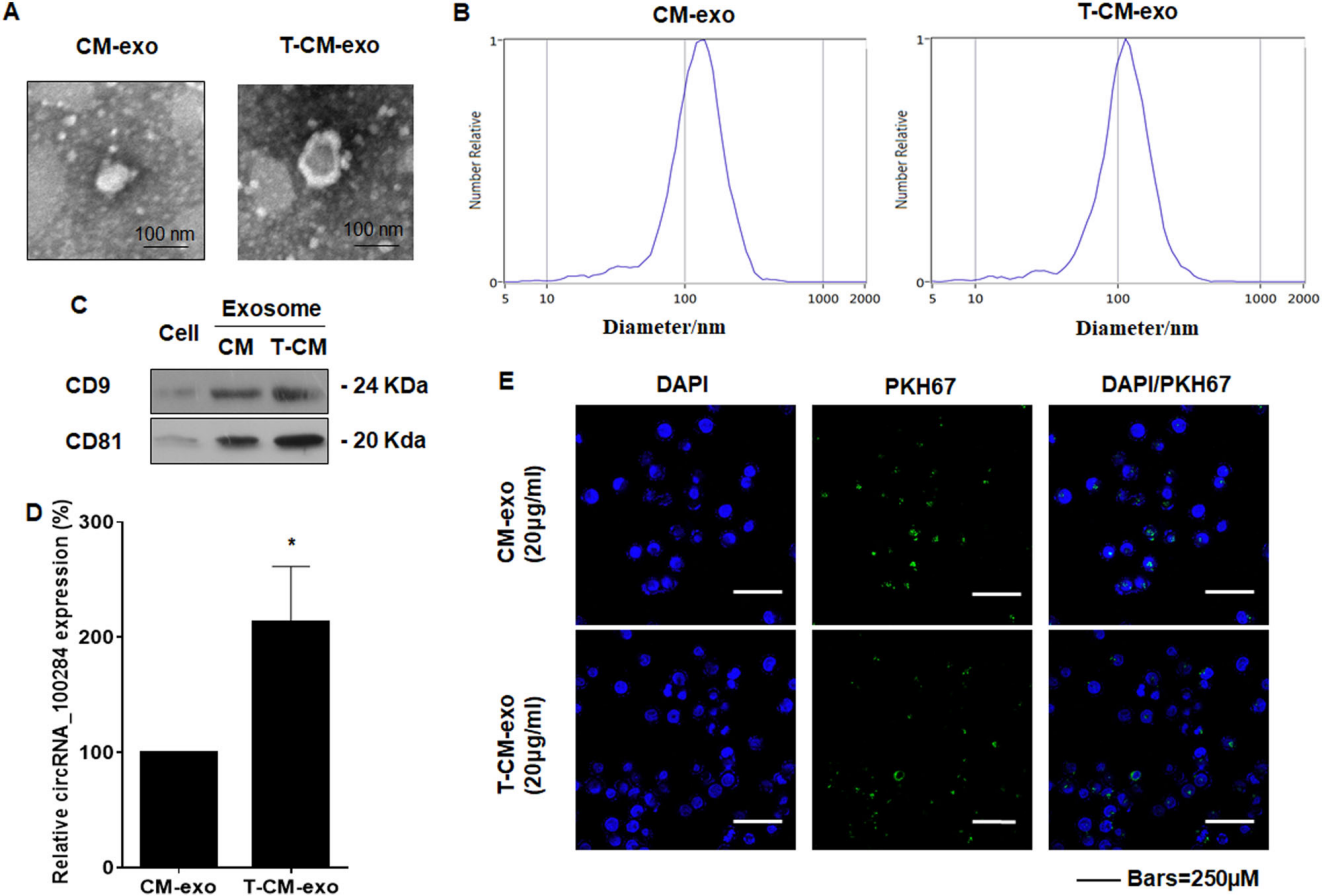
Transfer of circRNA_100284 derived from arsenite-transformed L-02 cells to normal L-02 cells via exosomes. *CM-exo* exosomes derived from passage-control L-02 cells, *T-CM-exo* exosomes derived from arsenite-transformed L-02 cells. Exosomes from C-L-02 cells and T-L-02 cells were fractionated by Exoquick. **a** Representative electron micrographs of CM-exo or T-CM-exo (right, bars = 100 nm). **b** Particle number and size analysis of CM-exo or T-CM-exo were determined by dynamic light scattering using a ZetaView® nanoparticle tracker (ParticleMetrix GmbH; Meerbusch, Germany). **c** Western blots of CD9 and CD81 in cells, CM-exo or T-CM-exo. **d** circRNA_100284 levels in CM-exo or T-CM-exo were measured by qRT-PCR (means ± SD, *n* = 3). *P < 0.05, different from CM-exo. CM-exo and T-CM-exo were labeled with PKH67, a green fluorescent cell linker. **e** Normal L-02 cells after 3 h incubation of exosomes with fluorescently labeled PKH67. Green represents CM-exo or T-CM-exo staining by PKH67, and blue represents nuclear DNA staining by DAPI

Exosomes, membrane-bound vessels that are released from cells, contain cargo including proteins, lipids, and RNA^19^. Thus, we predicted that circRNA_100284 is transferred from transformed cells into neighboring normal cells via exosomes. To establish the functions of exosomes, exosomal fractions were prepared from the culture media of passage-control L-02 cells and arsenite-transformed L-02 cells. Electron microscopy revealed that the exosomes of passage-control L-02 cells and arsenite-transformed L-02 cells showed typically rounded shapes (Fig. 3a). The particle sizes and counts of the exosomes showed no significant differences between exosomes derived from arsenite-transformed L-02 cells and passage-control L-02 cells as determined by a ZetaView® nanoparticle tracker (Fig. 3b). Western blot analysis confirmed the presence of exosome marker proteins CD81 and CD9^35^ in exosomes derived from arsenite-transformed L-02 cells and passage-control L-02 cells (Fig. 3c). The levels of circRNA_100284 were higher in exosomes from arsenite-transformed cells (Fig. 3d). Exosomes derived from passage-control L-02 cells or arsenite-transformed L-02 cells were labeled with a green fluorescent dye, PKH67. After 3 h of incubation with labeled exosomes, normal L-02 cells showed the intake of exosomes in the cytoplasm, as determined by fluorescence microscopy (Fig. 3e). Thus, the data show transfer of circRNA_100284 derived from arsenite-transformed L-02 cells into normal liver cells via exosomes.

### Exosomal circRNA_100284 derived from arsenite-transformed L-02 cells is involved in acceleration of the cell cycle in normal L-02 cells

**Fig. 4:**
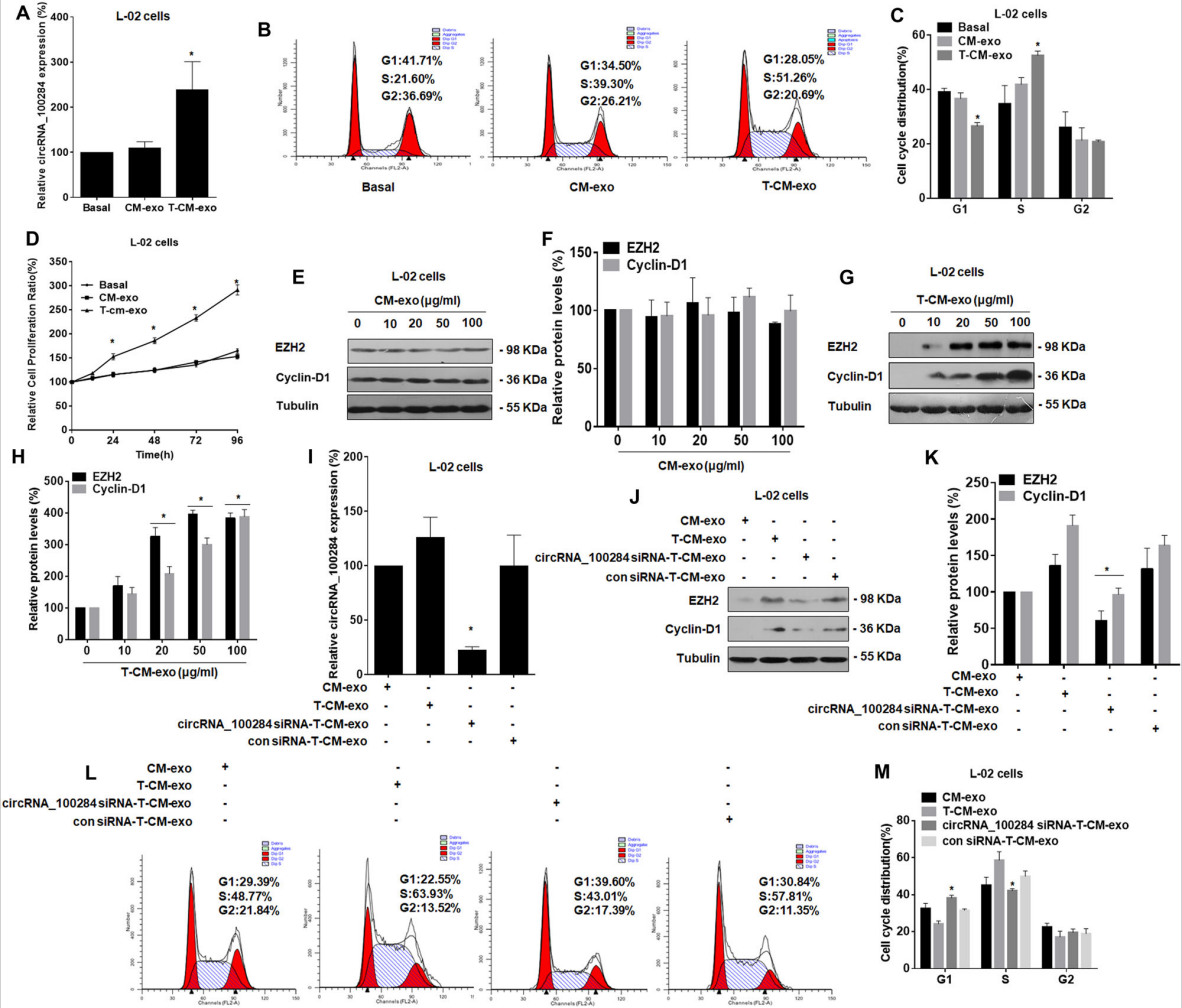
Exosomal circRNA_100284 derived from arsenite-transformed L-02 cells induces acceleration of the cell cycle and proliferation in normal L-02 cells. *CM-exo* exosomes derived from passage-control L-02 cells, *T-CM-exo* exosomes derived from arsenite-transformed L-02 cells. *circRNA_100284 siRNA-T-CM-exo*, exosomes derived from arsenite-transformed L-02 cells transfected with circRNA_100284 siRNA for 24 h; *con siRNA-T-CM-exo* exosomes derived from arsenite-transformed L-02 cells transfected with control siRNA for 24 h. Densities of bands were quantified by Image J software. Tubulin levels, measured in parallel, served as controls. Normal L-02 cells were treated with CM-exo or T-CM-exo for 24 h. **a** Levels of circRNA_100284 were determined by qRT-PCR assays (means ± SD, *n* = 3). **b** Flow cytometry was performed to analyze the cell cycle, and **c** representative histograms were prepared. **d** Proliferation efficiency was measured by use of a Cell Counting Kit-8 assay, and the relative ratios of cell proliferation were determined by comparison with cells in basal medium (means ± SD, *n* = 3). *P < 0.05, different from cells treated with CM-exo. Normal L-02 cells were treated with 0, 10, 20, 50, or 100 μg/mL CM-exo or T-CM-exo for 24 h. **e** and **g** western blots were performed; **f**, **h** relative protein levels (means ± SD, *n* = 3) of EZH2 and cyclin-D1 were determined in normal L-02 cells. *P < 0.05, different from control L-02 cells. Normal L-02 cells were treated with CM-exo, T-CM-exo, circRNA_100284-T-CM-exo, or con siRNA-T-CM-exo for 24 h. **i** The levels of circRNA_100284 were determined by qRT-PCR assays (means ± SD, *n* = 3). **j** Western blots were performed, and **k** relative protein levels (means ± SD, *n* = 3) of EZH2 and cyclin-D1 were determined. **l** Flow cytometry was performed to analyze the cell cycle, and **m** representative histograms were prepared. *P < 0.05, different from cells treated with T-CM-exo

To investigate the function of exosmal circRNA_100284, normal L-02 cells were exposed to exosomes derived from passage-control L-02 cells or arsenite-transformed L-02 cells. The levels of circRNA_100284 in cells treated with exosomes from arsenite-transformed L-02 cells were higher than that in cells treated with exosomes from passage-control L-02 cells (Fig. 4a). Exosomes derived from arsenite-transformed L-02 cells also accelerated the cell cycle (Figs. 4b, c) and promoted the proliferation at multiple time points (24, 48, 72, and 96 h) of normal L-02 cells (Fig. 4d). Exposure of L-02 cells to 0, 10, 20, 50, or 100 μg/mL of exosomes from arsenite-transformed L-02 cells for 24 h, but not from passage-control cells, induced increases of EZH2 and cyclin-D1 (Figs. 4e–h). Further, we used GW4869, which inhibits the secretion of exosomes^36^. Arsenite-transformed L-02 cells were treated with GW4869 (2.5 mM) or DMSO (0.005%) for 3 h. After exposure of arsenite-transformed L-02 cells to GW4869, the generation of exosomes was attenuated. Blocking of exosomes derived from arsenite-transformed L-02 cells inhibited the expression of circRNA_100284 (Fig. S3A), the expressions of EZH2 and cyclin-D1 (Fig. S3B and Fig. S3C), and the accelerated cell cycle (Fig. S3D and Fig. S3E) in L-02 cells.

In view of the findings described above, we presumed that exosomal circRNA_100284 derived from arsenite-transformed L-02 cells had an influence on the cell cycle and proliferation of normal L-02 cells. Normal L-02 cells were exposed to exosomes derived from arsenite-transformed L-02 cells transfected with or without circRNA_100284 siRNA. The higher levels of circRNA_100284 in normal L-02 cells were reduced by exosomes from arsenite-transformed L-02 cells transfected with circRNA_100284 siRNA (Fig. 4i). Knockdown of circRNA_100284 blocked the increases of protein levels of EZH2 and cyclin-D1 induced by exosomes from arsenite-transformed L-02 cells (Figs. 4j,k). Moreover, flow cytometry showed that the accelerated G1/S transition induced by exosomes from arsenite-transformed L-02 cells was reduced after exposure to exosomes from arsenite-transformed L-02 cells transfected with circRNA_100284 siRNA (Figs. 4l,m). Thus, in normal L-02 cells, exosomal circRNA_100284 derived from arsenite-transformed L-02 cells is involved in increases of EZH2 and cyclin-D1 and in an abnormal cell cycle.

### Exosomal circRNA_100284 derived from arsenite-transformed L-02 cells induces the acceleration of the cell cycle and promoted proliferation via miR-217 in normal L-02 cells

**Fig. 5:**
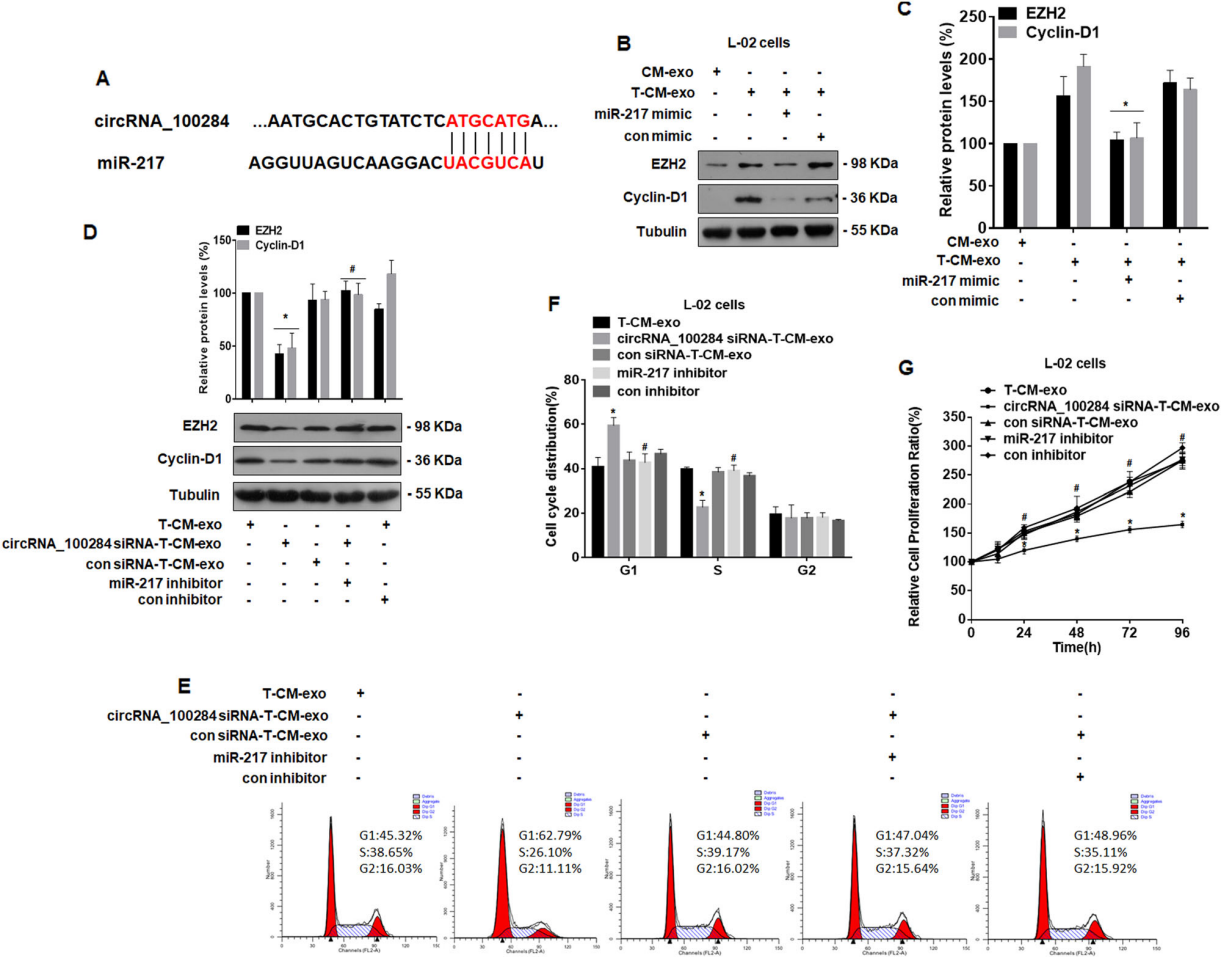
Exosomal circRNA_100284 derived from arsenite-transformed L-02 cells induces acceleration of the cell cycle and promotes proliferation via miR-217 in normal L-02 cells. *T-CM-exo* exosomes derived from arsenite-transformed L-02 cells, *CircRNA_100284 siRNA-T-CM-exo* exosomes derived from arsenite-transformed L-02 cells transfected with circRNA_100284 siRNA for 24 h, *con siRNA-T-CM-exo* exosomes derived from arsenite-transformed L-02 cells transfected with control siRNA for 24 h. Densities of bands were quantified by Image J software. Tubulin levels, measured in parallel, served as controls. **a** A schematic graph illustrating binding sites between circRNA_100284 and miR-217. After normal L-02 cells were transfected with control mimic or miR-217 mimic for 24 h, they were treated with CM-exo or T-CM-exo for 24 h. **b** Western blots were performed, and **c** relative protein levels (means ± SD, *n* = 3) of EZH2 and cyclin-D1 were determined. *P < 0.05, different from cells treated with T-CM-exo. After normal L-02 cells were transfected with control inhibitor or miR-217 inhibitor for 24 h, they were treated with T-CM-exo, circRNA_100284-T-CM-exo, or con siRNA-T-CM-exo for 24 h. **d** Western blots were performed, and relative protein levels (means ± SD, *n* = 3) of EZH2 and cyclin-D1 were determined. **e** Flow cytometry was performed to analyze the cell cycle, and **f** representative histograms were prepared. **g** Proliferation efficiency was measured by use of the Cell Counting Kit-8 assay, and the relative ratios of cell proliferation were determined by comparison with cells in basal medium (means ± SD, *n* = 3). *P < 0.05, different from cells treated with T-CM-exo, ^*#*^*P* < 0.05, different from cells treated with circRNA_100284-T-CM-exo alone

circRNAs usually regulate gene expression by acting as miRNA sponges^37^. Since, in a previous study, we found that, in HaCaT cells, circRNA_100284 is involved in the arsenite-promoted cell cycle via miR-217^15^, we predicted that the pathway was also involved in the abnormal cell cycle of L-02 cells induced by exosomes from arsenite-transformed L-02 cells. Binding sites between circRNA_100284 and miR-217 (Fig. 5a) were analyzed by the bioinformatics methods, circBase (http://circrna.org/) and CircInteractome (http://circinteractome.nia.nih.gov/). Normal L-02 cells were transfected with a control or an miR-217 mimic and then exposed to exosomes derived from arsenite-transformed L-02 cells. Overexpression of miR-217 attenuated the upregulation of EZH2 and cyclin-D1 induced by exosomes derived from arsenite-transformed L-02 cells (Figs. 5b,c). Next, normal L-02 cells were transfected with a control or an miR-217 inhibitor and then exposed to exosomes derived from arsenite-transformed L-02 cells transfected with or without circRNA_100284 siRNA. Western blot analyses revealed that the miR-217 inhibitor reversed the effects of exosomes from arsenite-transformed L-02 cells transfected with circRNA_100284 siRNA on the levels of EZH2 and cyclin-D1 in normal L-02 cells (Fig. 5d). In addition, flow cytometry showed that the inhibited G1/S transition induced in normal L-02 cells by exosomes from arsenite-transformed L-02 cells transfected with circRNA_100284 siRNA was also restored by an miR-217 inhibitor (Figs. 5e,f). Moreover, exosomes derived from cells transfected with circRNA_100284 siRNA inhibited proliferation of normal L-02 cells at multiple time points (24, 48, 72, and 96 h), but this effect was reversed by an miR-217 inhibitor (Fig. 5g). To conclude, in normal liver cells, exosomal cicrRNA_100284 accelerates the cell cycle and promotes cell proliferation via miR-217.

### Exosomal circRNA_100284 derived from arsenite-transformed L-02 cells is involved in the malignant transformation of non-transformed L-02 cell

**Fig. 6:**
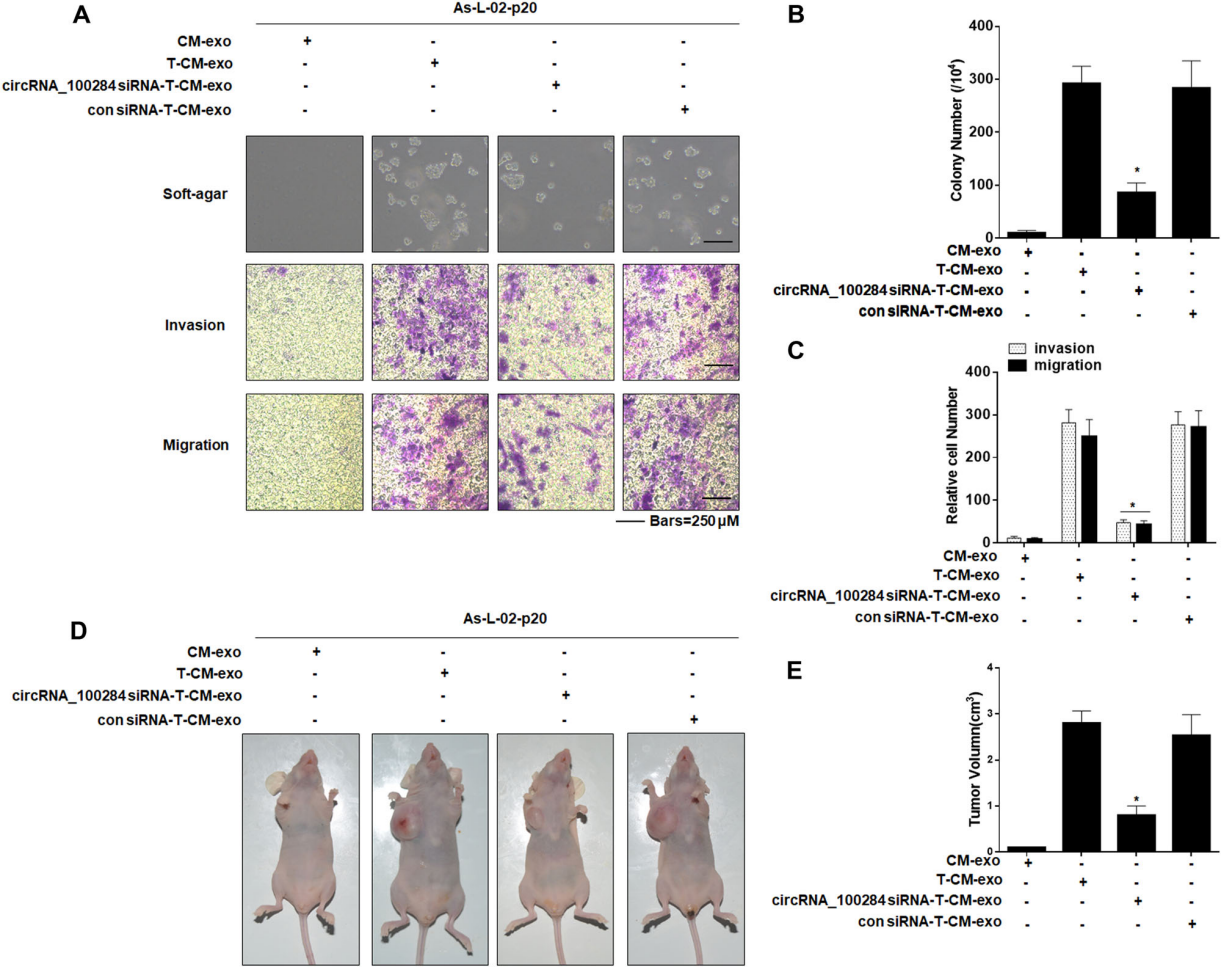
Effects of exosomal circRNA_100284 on the process of malignant transformation of As-L-02-p20 cells. *As-L-02-p20 cells* L-02 cells exposed to 2 μM of arsenite for 20 passages, *CM-exo* exosomes derived from passage-control L-02 cells, *T-CM-exo* exosomes derived from arsenite-transformed L-02 cells. *circRNA_100284 siRNA-T-CM-exo*, exosomes derived from arsenite-transformed L-02 cells transfected with circRNA_100284 siRNA for 24 h; *con siRNA-T-CM-exo* exosomes derived from arsenite-transformed L-02 cells transfected with control siRNA for 24 h. As-L-02-p20 cells were treated with CM-exo, T-CM-exo, circRNA_100284-T-CM-exo, or con siRNA-T-CM-exo for 24 h. **a** Colony formation in soft agar (upper, bars = 250 μm) and Transwell assays (lower, bars = 250 μm). **b**, **c** Relative colony numbers and relative levels of cell invasion and migration were determined (means ± SD, *n* = 3). **d** Tumors were examined, and **e** their volumes were measured (means ± SD, *n* = 6) at 4 weeks after cells were inoculated into nude mice. *P < 0.05, different from As-L-02-p20 cells treated with T-CM-exo

To determine whether exosomal circRNA_100284 derived from arsenite-transformed L-02 cells was involved in the malignant transformation of non-transformed L-02 cells, we treated As-L-02-p20 cells with exosomes derived from normal L-02 cells or arsenite-transformed L-02 cells transfected with or without circRNA_100284 siRNA. Anchorage-independent growth showed that As-L-02-p20 cells treated with exosomes derived from arsenite-transformed L-02 cells formed numerous colonies, but those treated with exosomes derived from arsenite-transformed L-02 transfected with circRNA_100284 formed only a few colonies, and these were of small size (Fig. 6a, upper, and Fig. 6b). To determine whether exosomal circRNA_100284 facilitated migration and invasion of L-02 cells, invasion through Matrigel and migration through Transwells were evaluated. Relative to controls, inhibition of exosomal circRNA_100284 impeded the enhanced invasion and migration of L-02 cells treated with exosomes derived from arsenite-transformed L-02 cells (Fig. 6a, lower, and Fig. 6c). Moreover, As-L-02-p20 cells treated with circRNA_100284-knockdown exosomes derived from arsenite-transformed L-02 cells were injected into nude mice. Inhibition of exosomal circRNA_100284 decreased the sizes of tumors that developed relative to the group treated with exosomes from arsenite-transformed cells (Figs. 6d, 6e). These results confirmed that exosomal circRNA_100284 derived from arsenite-transformed L-02 cells was involved in the malignant transformation of non-transformed L-02 cells.

### Levels of circulating exosomal circRNA_100284 are overexpressed in people exposed to arsenite

**Fig. 7:**
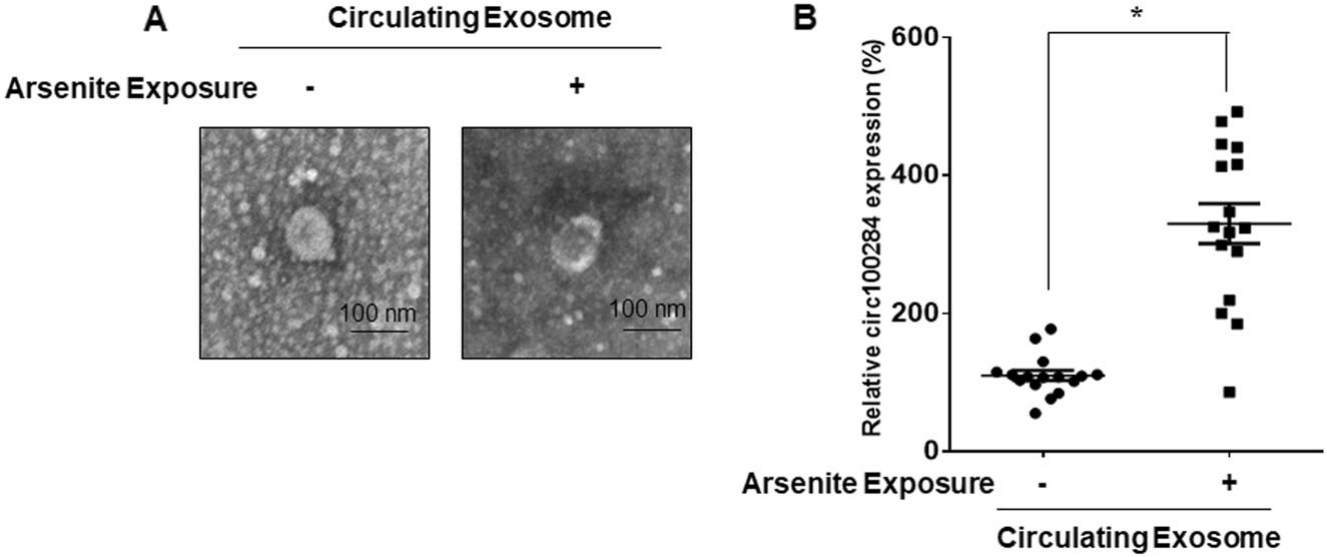
Levels of circulating exosomal circRNA_100284 are overexpressed in people exposed to arsenite. *Circulating Exosome* exosomes isolated from the sera of individuals exposed or not exposed to arsenite. **a** Representative electron micrograph of circulating exosomes (bars = 100 nm). **b** The levels of circRNA_100284 in circulating exosomes were determined by qRT-PCR assays (means ± SD, *n* = 16). *P < 0.05, different from circulating exosomes isolated from the sera of individuals not exposed to arsenite

Exosomes are considered as biomarkers of diseases owning to their capacity of transferring their cargo RNA molecules, such as mRNAs, miRNAs^19^, and circRNAs^24^. To determine whether circRNA_100284 was involved in the response to arsenite exposure, exosomes were isolated from the sera of people exposed or not exposed to arsenite. Electron microscopic examination showed that purified exosomes presented a size distribution consistent with exosome vesicles (30–100 nm, Fig. 7a). There was higher expression of exosomal circRNA_100284 in the sera of people exposed to arsenite compared with that in sera of paired healthy volunteers (Fig. 7b). These results indicated that exosomal circRNA_100284 can be used as a biomarker of arsenite exposure.

## Discussion

circRNAs are noncoding RNAs that are widespread and diverse in eukaryotic cells. Compared with linear RNA, circRNAs have noncanonical splicing without a free 3’ or 5’ end^38^. The functions of circRNAs in various cancers are being elucidated. For example, Hsa_circ_0001649 and hsa_circ_0005075 are potential diagnostic biomarkers for hepatocellular carcinoma (HCC). In our previous study, we found, by a circRNA microarray, that circRNA_100284 showed the greatest upregulation in arsenite-transformed HaCaT cells and that, in human keratinocytes, it was involved in the accelerated cell cycle induced by arsenite in the process of carcinogenesis^15^. In the present study, we found that, in normal L-02 cells, circRNA_100284 was upregulated after both acute and chronic exposure to arsenite.

Long-term exposure to low concentrations of arsenite contributes to the malignant transformation of various cell lines^25,39,40^. In the present study, L-02 cells were continuously exposed to 2 μM arsenite for 30 passages. Low concentrations of arsenite enhanced neoplastic transformation of cells, as determined by anchorage-independent growth in soft agar and tumorigenesis in nude mice. However, inhibition of circRNA_100284 led to fewer colonies, less invasion, and less migration compared to control arsenite-transformed L-02 cells; overexpression of circRNA_100284 in non-transformed cells showed the opposite results. These results indicate that circRNA_100284 is involved in the malignant transformation induced by arsenite in normal epithelial cells.

In the tumor microenvironment, complex mechanisms are involved in tumor progression, particularly in intercellular communication between malignant cells and nonmalignant cells^41^. In our previous study, we found that the medium from transformed HBE cells affects physiological and pathological processes in normal HBE cells^22,42^. On the basis of our previous results and those of others, we hypothesized that arsenite-transformed cells secrete molecules to affect the surrounding environment, leading to the malignancy of neighboring normal cells. In the present study, we observed upregulated levels of circRNA_100284 in normal L-02 cells treated with medium from arsenite-transformed L-02 cells, along with an accelerated cell cycle and increased proliferation. These results confirmed our assumption that a cell cycle change and higher proliferation are promoted by molecules secreted into the medium by transformed cells.

The transmitters of cell–cell interactions can be secreted proteins, ions, or exosomes. Exosomes, derived from the endosomal compartment, are released in the extracellular milieu under various physiological and pathological conditions^43^. In the present study, we showed that exosomes from arsenite-transformed L-02 cells, confirmed by electron microscopy and transferred to the surrounding cells, are transmitters of cell–cell communication.

RNA isolated from exosome preparations contains circRNAs^44^. In the present study, we found that the levels of circRNA_100284 were increased in exosomes derived from arsenite-transformed L-02 cells. Moreover, exosomes derived from arsenite-transformed L-02 cells elevated the levels of circRNA_100284 and promoted the cell cycle and proliferation of normal L-02 cells. When exposed to exosomes derived from arsenite-transformed L-02 cells, the expression of EZH2, a potential biomarker of proliferation^45,46^, and cyclin-D1, which regulates the G1 to S phase transition in the cell cycle^47,48^, increased in normal L-02 cells. There was no such change in normal cells treated with exosomes from passage-control L-02 cells. These changes were inhibited when the generation of exosomes was inhibited by GW4869 or the expression of circRNA_100284 was blocked in arsenite-transformed L-02 cells. The results indicated that circRNA_100284, shuttled from arsenite-transformed L-02 cells to normal L-02 cells via exosomes, induced an accelerated cell cycle and promoted proliferation of normal cells. Abnormal cell proliferation resulting from deregulated cell cycle progression is involved in neoplastic transformation^32^. In the present study, L-02 cells exposed to arsenite for 20 passages formed more colonies, showed enhanced invasion and migration, and induced tumors in nude mice after treatment with exosomes derived from arsenite-transformed cells compared to passage-control cells. These results were reversed when the expression of circRNA_100284 was blocked in arsenite-transformed L-02 cells. These effects indicated that exosomal circRNA_100284 may lead to the malignant transformation of non-transformed L-02 cells.

miRNAs are involved in regulation of the cell cycle and proliferation of HCC cells^49,50^. We previously reported that, for arsenite-transformed HaCat cells, circRNA_100284 regulates the cell cycle by acting as a sponge of miR-217^15^, a tumor suppressor that regulates cell proliferation in various cancers, including HCCs^51^. miR-217 inhibits cell proliferation through inducing arrest in the G2/M phase of the cell cycle^52^. The present results showed that overexpression of miR-217 in normal cells reduced the increased levels of EZH2 and cyclin-D1 induced by exosomes from arsenite-transformed L-02 cells. An miR-217 inhibitor reversed the effects, including the decreased levels of EZH2 and cyclin-D1, and inhibited the cell cycle and proliferation caused by exosomes from arsenite-transformed L-02 cells transfected with circRNA_100284 siRNA. These results show that exosomal circRNA_100284 regulates the cell cycle and cell proliferation via miR-217. In cancers, miR-217 targets EZH2 (refs.^53,54^). However, the mechanism for regulation of EZH2 by miR-217 in L-02 cells and how it affects malignant transformation remain to be determined.

In Guizhou Province, China, food contamination and air pollution caused by coal burning have been linked to severe arsenite exposure^55,56^. In the present study, blood samples were collected from patients exposed to arsenite and from healthy volunteers in Guizhou province. Exosomes were isolated from the sera. Arsenite exposure induced an increase of circRNA_100284 levels in circulating exosomes. Thus, exosomal circRNA_100284 may be a biomarker for diagnosis of arsenicosis.

**Fig. 8:**
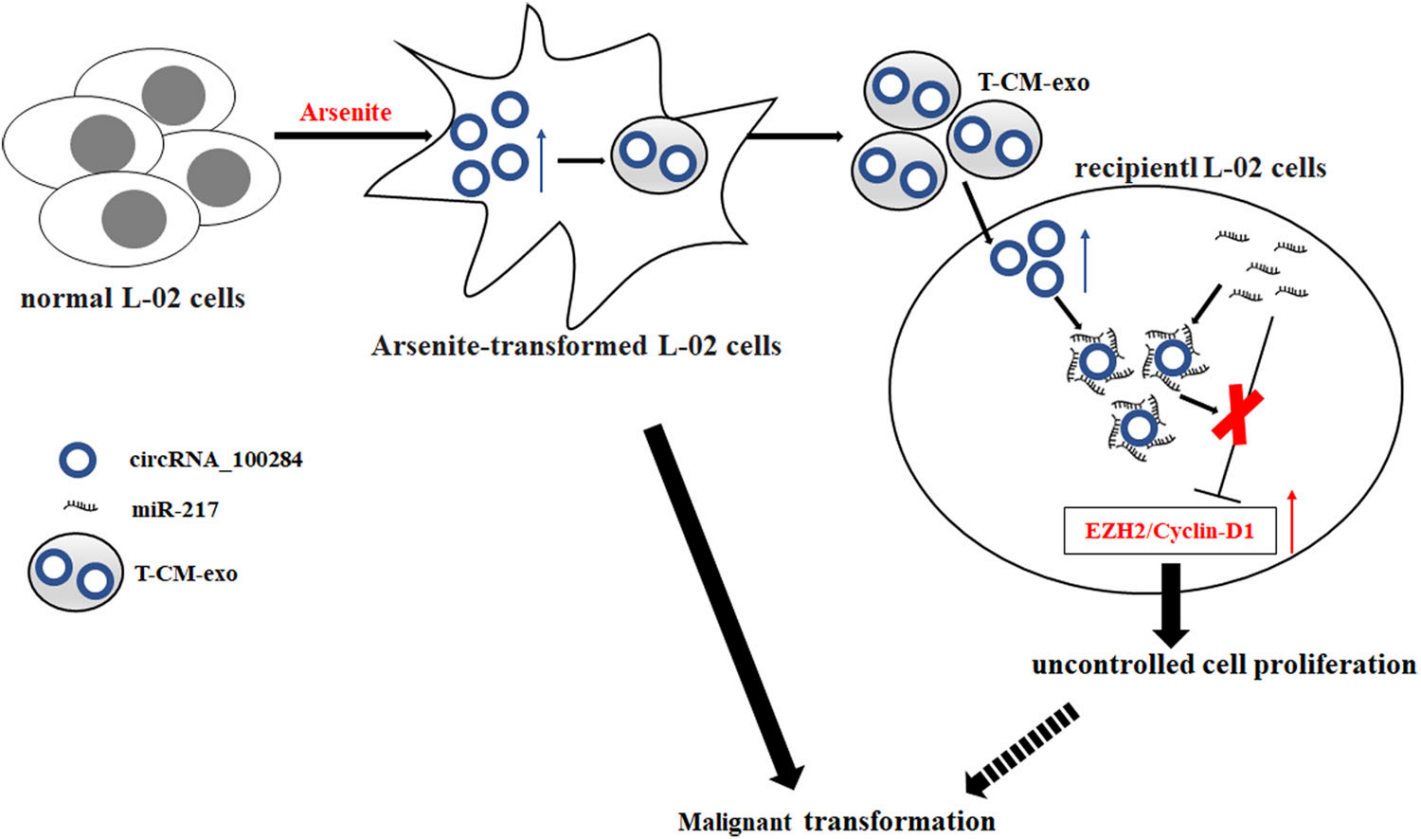
A schematic representation of the proposed pathway by which exosomal circRNA_100284 causes proliferation of L-02 cells in arsenite carcinogenesis. Chronic exposure to arsenite elevates circRNA_100284 levels, which is involved in the malignant transformation of normal L-02 cells. circRNA_100284 from arsenite-transformed cells transferred into normal L-02 cells via exosomes induce acceleration of the cell cycle and promotes proliferation via miR-217

To conclude, long-term exposure to arsenite induced the malignant transformation of L-02 cells and overexpression of circRNA_100284, which could be shuttled from transformed L-02 cells to normal cells via exosomes. In normal cells, exosomal circRNA_100284 induced an accelerated cell cycle and promoted proliferation via acting with miR-217 (Fig. 8). These changes are apparently involved in arsenite-induced malignant transformation. In sum, the results demonstrate that exosomal circRNAs act as messengers in cell–cell communication and suggest a mechanism for carcinogenesis induced by arsenite.

## Electronic supplementary material

SUPPLEMENTAL MATERIAL
