# Supplementary material for: Exosomal circRNA_100284 from arsenite-transformed cells, via microRNA-217 regulation of EZH2, is involved in the malignant transformation of human hepatic cells by accelerating the cell cycle and promoting cell proliferation
Source: Cell Death Dis. 2018 Apr 19;9(5):454. doi: 10.1038/s41419-018-0485-1 (PMC5908808; doi:10.1038/s41419-018-0485-1)
Supplement: Supplementary file 1 — SUPPLEMENTAL MATERIAL [file 41419_2018_485_MOESM1_ESM.docx]

***Supplementary*** ***Figures and Figure Legends***

***
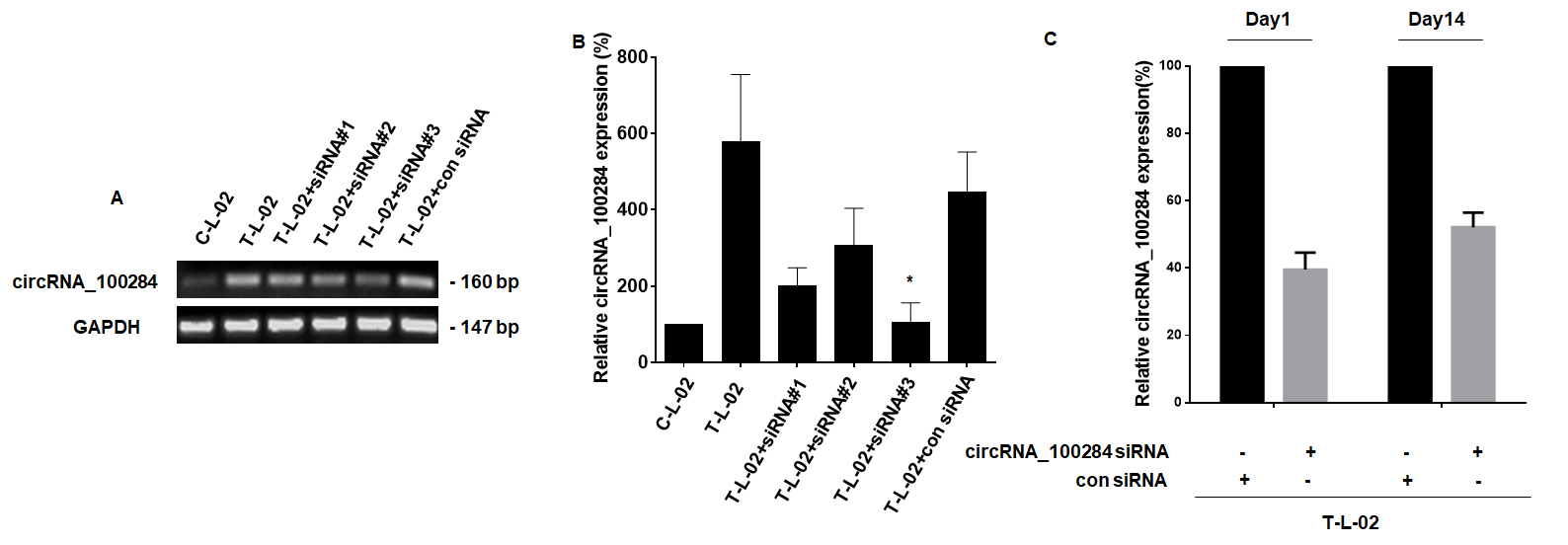
***

***Fig. S1.*** ***Knockout efficiency of three circRNA_100284 siRNAs in arsenite-transformed L-02 cells.***

*C-L-02*, passage-control L-02 cells; *T-L-02*, arsenite-transformed L-02 cells. T-L-02 cells were transfected with control siRNA or with one of three circRNA_100284 siRNAs for 24 h. Levels of circRNA_100284 were determined by (**A**) RT-PCR (means ± SD, n = 3) and (**B**) quantitative RT-PCR (means ± SD, n = 3), *^*^P* < 0.05, different from T-L-02 cells in the absence of circRNA_100284 siRNA#3. (**C**) To examine the efficiency of inhibition of circRNA_100284 siRNA, RT-PCR was performed to evaluate the expression of circRNA_100284 at days 1 and 14 (means ± SD, n = 3), *^*^P* < 0.05, different from T-L-02 cells treated with control siRNA.


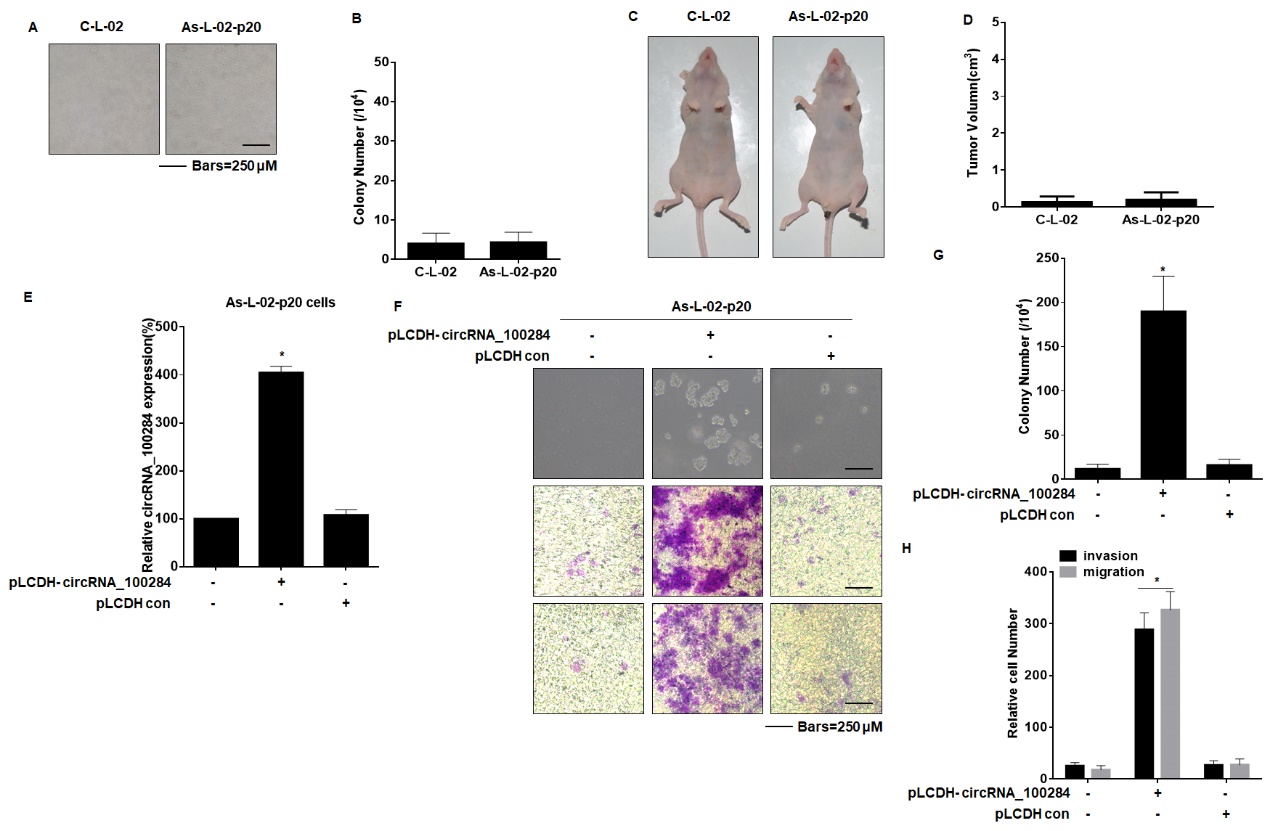


***Fig. S2. circRNA_100284 are overexpressed in As-L-02-p20 cells.***

*C-L-02*, passage-control L-02 cells; *As-L-02-p20*, L-02 cells exposed to 2 μM arsenite for 20 passages. (A) Colonies and (B) their numbers (means ± SD, n = 3) of C-L-02 cells and As-L-02-p20 cells in soft agar (bars = 250 μm). (C) Tumors were examined, and (D) their volumes were measured (means ± SD, n = 6) at 4 weeks after C-L-02 cells and As-L-02-p20 cells were inoculated into nude mice. T-L-02 cells were transfected with 10 nM pLCDH control or 10 nM pLCDH-circRNA_100284 for 24 h. (**E**) The levels of circRNA_100284 were determined by qRT-PCR assays (means± SD, n =3). (**F**) Colony formation in soft agar (upper, bars = 250 μm) and Transwell assays (lower, bars = 250 μm). (**G**) and (**H**) Relative colony numbers and relative levels of cell invasion and migration were determined (means ± SD, n = 3). ^*^P < 0.05, different from As-L-02-p20 cells.


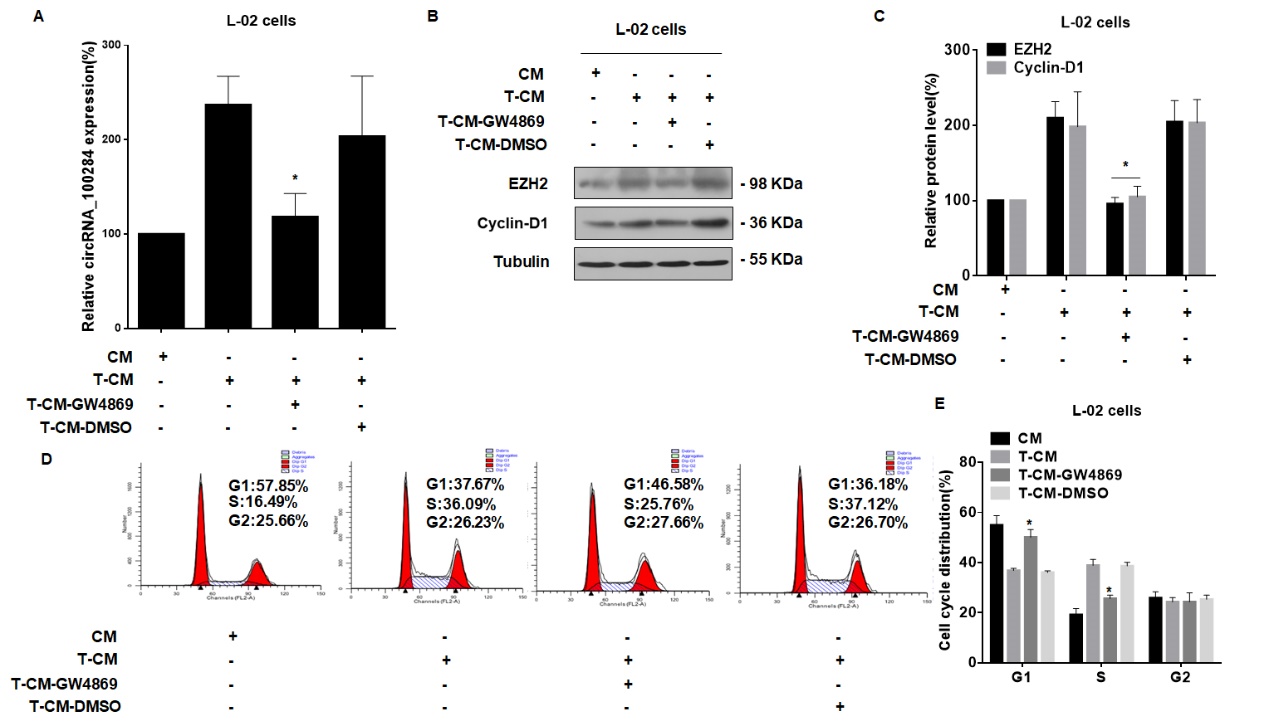


***Fig. S3. Blocking of exosomes derived from arsenite-transformed L-02 cells inhibits the expression of circRNA_100284 and the acceleration of cell cycle in normal L-02 cells.***

*CM*, medium from passage-control L-02 cells; *T-CM*, medium from arsenite-transformed L-02 cells; *T-CM-GW4869*, medium from arsenite-transformed L-02 cells treated with 2.5 mM GW4869; *T-CM-DMSO*, medium from arsenite-transformed L-02 cells treated with 0.005% DMSO. Densities of bands were quantified by Image J software. Tubulin levels, measured in parallel, served as controls. L-02 cells were treated with CM, T-CM, T-CM-GW4869, or T-CM-DMSO for 24 h. (**A**) circRNA_100284 levels in L-02 cells were determined by qRT-PCR assays (means± SD, n =3). (**B**) Western blots were performed, and (**C**) relative protein levels (means± SD, n =3) of EZH2 and cyclin-D1 were determined. (**D**) Flow cytometry was performed to analyze the cell cycle, and (**E**) representative histograms were prepared. **P* < 0.05, different from cells treated with the medium from arsenite-transformed L-02 cells alone.
